# Supplementary material for: The causal relationship between the human gut microbiota and pyogenic arthritis: a Mendelian randomization study
Source: Front Cell Infect Microbiol. 2024 Nov 26;14:1452480. doi: 10.3389/fcimb.2024.1452480 (PMC11629706; doi:10.3389/fcimb.2024.1452480)
Supplement: Supplementary file 1 [file DataSheet1.zip › mendelian test/ebi-a-GCST90027486.csv_leave_one_out.pdf]

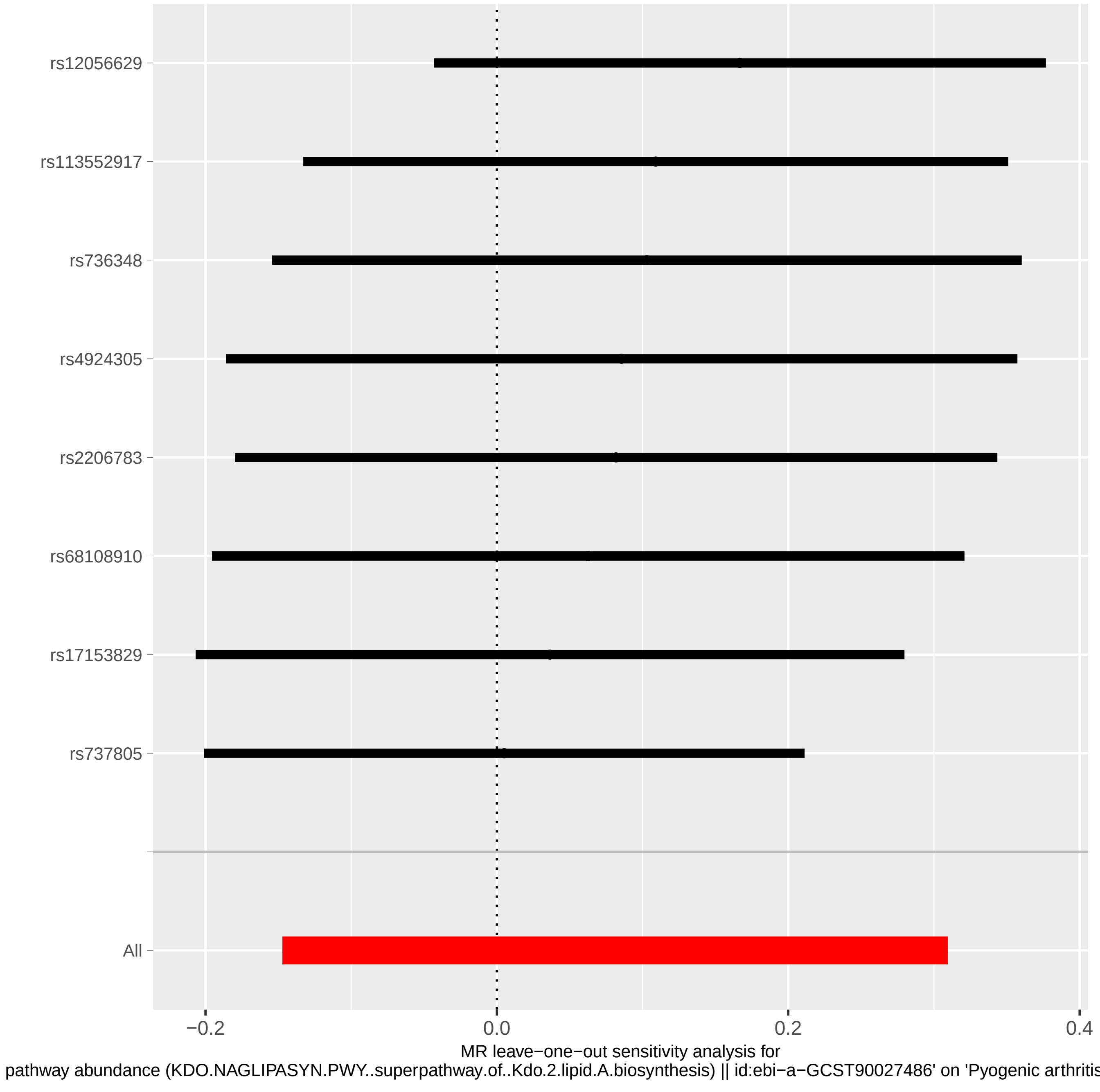

rs12056629
rs113552917
rs736348
rs4924305
rs2206783
rs68108910
rs17153829
rs737805
All
−0.2
0.0
0.2
0.4
MR leave−one−out sensitivity analysis for
pathway abundance (KDO.NAGLIPASYN.PWY..superpathway.of..Kdo.2.lipid.A.biosynthesis) || id:ebi−a−GCST90027486' on 'Pyogenic arthritis
